# Preparation, Physicochemical Properties, and Hemocompatibility of the Composites Based on Biodegradable Poly(Ether-Ester-Urethane) and Phosphorylcholine-Containing Copolymer

**DOI:** 10.3390/polym11050860

**Published:** 2019-05-11

**Authors:** Jun Zhang, Bing Yang, Qi Jia, Minghui Xiao, Zhaosheng Hou

**Affiliations:** 1 College of Chemistry, Chemical Engineering and Materials Science, Shandong Normal University, Jinan 250014, China; zj971127@163.com (J.Z.); xiaominghui98@163.com (M.X.); 2 Key Laboratory of Public Security Management Technology in Universities of Shandong, Shandong Management University, Jinan 250357, China; yb197325@163.com; 3 Qilu Pharmaceutical Co. Ltd., Jinan 250104, China; haperwork@163.com

**Keywords:** poly(ether-ester-urethane), MPC copolymers, composites, physicochemical properties, hemocompatibility

## Abstract

To improve the hemocompatibility of the biodegradable medical poly(ether-ester-urethane) (PEEU), containing uniform-size aliphatic hard segments that was prepared in our lab, a copolymer containing phosphorylcholine (PC) groups was blended with the PEEU. The PC-copolymer of poly(MPC-co-EHMA) (PMEH) was first obtained by copolymerization of 2-methacryloyloxyethyl phosphorylcholine (MPC) and 2-ethylhexyl methacrylate (EHMA), and then dissolved in mixed solvent of ethanol/chloroform to obtain a homogeneous solution. The composite films (PMPU) with varying PMEH content were prepared by solvent evaporation method. The physicochemical properties of the composite films with varying PMEH content were researched. The PMPU films exhibited higher thermal stability than that of the pure PEEU film. With the PMEH content increasing from 5 to 20 wt%, the PMPU films also possessed satisfied tensile properties with ultimate stress of 22.9–15.8 MPa and strain at break of 925–820%. The surface and bulk hydrophilicity of the films were improved after incorporation of PMEH. In vitro degradation studies indicated that the degradation rate increased with PMEH content, and it took 12–24 days for composite films to become fragments. The protein adsorption and platelet-rich plasma contact tests were adapted to evaluate the surface hemocompatibility of the composite films. It was found that the amount of adsorbed protein and adherent platelet on the surface decreased significantly, and almost no activated platelets were observed when PMEH content was above 5 wt%, which manifested good surface hemocompatibility. Due to the biodegradability, acceptable tensile properties and good surface hemocompatibility, the composites can be expected to be applied in blood-contacting implant materials.

## 1. Introduction

Polyurethanes (PUs) are a kind of polymer with carbamate groups (–NHCOO–) on the backbones. Compared with other biomedical materials, PUs possess a multitude of advantages, including high tenacity, chemical resistance, and adjustable mechanical flexibility [1,2,3]. Due to their excellent physical-mechanical properties and adequate biocompatibility, PUs have been widely used in the medical field for almost half a century as heart valves, pacemaker wires, vascular grafts, cardioids, artificial skin joints, and catheters [4,5,6,7]. 

Biodegradable PUs are designed to undergo hydrolytic degradation to produce noncytotoxic products [8]. Most PUs based on aliphatic diisocyanate are prepared for biomedical application because they have lower toxicity compared to aromatic ones [9,10]. However, because of the absence of hard segments of significant length, these kinds of PUs exhibit dissatisfactory tensile properties like low tensile strength [11]. Penning and coworkers found that PUs containing long uniform-size hard segments possess excellent tensile properties [12]. In our previous reports [13,14], several kinds of biodegradable medical PUs based on aliphatic diurethane diisocyanate were prepared. The uniform chemical structure of hard segments improves the microphase separation degree of soft and hard segments, and, at the same time, the denser hydrogen bonds among carbamate units give a more compact physical-linking network structure, leading to comparative or even better tensile properties than aromatic PUs.

Biocompatibility, especially hemocompatibility, is another important requirement for the clinical implant or/and blood-contacting materials [15,16,17,18]. When PUs are used as long-term blood-contacting materials, proteins can accumulate rapidly on the material surface, subsequently platelets are activated, and then blood coagulation and thrombus occur [19,20]. Therefore, many strategies have been developed in order to achieve improved hemocompatibility of PUs. The most widely used method is chemical surface modification [21,22,23], which mainly includes introduction a high activation barrier to repel proteins by grafting hydrophilic polymers or biomimicking materials on the surface. However, the surface modification approaches inevitably deteriorate the bulk properties, especially the tensile properties of the substrates, which is difficult to overcome.

Recently, much attention has been concentrated on synthetic/natural polymer composites because it is simple and efficient to obtain new materials from mixing two different polymer materials. In the previous articles [24,25,26], the composites of PU and bioactive polymers were prepared and their fundamental properties were evaluated. The hemocompatibility of the composites, with attention to protein adsorption and platelet adhesion, was much better than that of original PU material. In addition, the tensile properties had no obvious change after addition a small quantity of bioactive polymer to PU. It provides a novel technique for exploitation of PU in biomedical application.

Phosphorylcholine (PC) is a hydrophilic zwitterionic head group of cell membrane phosphatidylcholine that endows the cell membrane with ideal biocompatibility, especially in resistance to protein adsorption and platelet adhesion [27,28]. In order to exploit new biocompatible materials, the methacrylate monomer-bearing PC group 2-methacryloyloxyethyl phosphorylcholine (MPC) was synthesized by Nakabayashi [29], and later Chapman [30]. Research on surface modification of biomaterials with PC functionality has been developed rapidly after publication of the MPC [31,32]. Because of the polymerizable methacrylate moiety, MPC can easily be copolymerized with other monomers to enable the design of numerous materials with various molecular architectures. The modification of biomedical PU with the MPC copolymer by blending to improve the biocompatibility has been studied by many researchers for nearly thirty years [33,34,35]. For example, Ishihara groups [36,37] prepared the composite films of MPC polymer and commercial PU (Tecoflex^®^ and Pellethane^®^ 2363-90), and the film materials exhibited excellent nonthrombogenicity in contact with human whole blood. The PC groups could be concentrated effectively near the surface in the plasma resulting in the formation of a ‘self-assembled biomimetic membrane bilayers’, which can form a thermodynamic hydration barrier over the surface and suppress any unfavorable interaction with blood cells and proteins. Therefore, it can be hypothesized that incorporation of MPC polymers will improve the biocompatibility (especially hemocompatibility) of new kinds of medical PU with uniform-size hard segments prepared in our lab. 

In this paper, the composites of the PU–PC copolymer were prepared by simple physical blending to improve the hemocompatibility of PU materials. The biodegradable poly(ether-ester-urethane) (PEEU), which contains uniform-size hard segments, was obtained in our lab and used as model PU. Based on the fundamental properties of PC-containing polymers, the MPC copolymer was used as a polymeric additive, which could blend and interact with PEEU. The effect of the MPC copolymer introduced in the PEEU films on the physicochemical properties of PEEU was investigated. Furthermore, the surface hemocompatibility of the composite films was evaluated by protein adsorption and platelet adhesion tests.

## 2. Materials and Methods

### 2.1. Materials

MPC (>98%) were purchased from J&K Scientific Ltd. (Beijing, China) and used without further purification. 2-Ethylhexyl methacrylate (EHMA, Shanghai Macklin Biochemical Co., Ltd., Shanghai, China) was dried with anhydrous magnesium sulfate and distilled under reduced pressure, then saved at −20 °C before use. 2,2′-Azobisisobutyronitrile (AIBN) was obtained from Shanghai Macklin Biochemical Co., Ltd. (Shanghai, China) and recrystallized several times from ethanol. Poly (ethylene glycol) (PEG, *M*_n_ = 600, Aladdin Reagent Co., Ltd., China) was dehydrated at 110 °C for ~4 h under vacuum. l-lactide (l-LA, J&K Scientific Co., Ltd.) was recrystallized several times times from dry ethyl acetate. ε-Caprolactone (ε-CL, Sigma-Aldrich, city, country) was distilled from CaH_2_ under reduced pressure. *N*,*N*-dimethylformamide (DMF, Beijing Chemical Reagent Co., Ltd., Beijing, China) was refluxed with phosphorus pentoxide for about 5 h and then distilled under reduced pressure. Diurethane diisocyanate (hexanediisocyanate-1,4-butanediol-hexanediisocyanate, HBH) was synthesized in our lab according to our previous paper [13]; NMR and HRMS analyses were used to confirme its chemical structure. Phosphate buffer saline (PBS, pH = 7.4) was supplied by Beijing Chemical Reagent Co., Ltd. and used as received. Other regents were AR grade and purified by standard methods.

### 2.2. Preparation of PEEU

The PEEU was prepared referring to our published literature according to Figure S1 [13]. In brief, ε-CL (0.15 mol), l-LA (0.19 mol), and PEG600 (0.05 mol) were mixed in a vacuum flask under dried argon atmosphere. After three rounds of deoxygenation, catalyst stannous octoate (0.1 wt% of monomers) was added and the reaction was carried out at 140 °C for 36 h under vacuum to obtain the prepolymer (Figure S1a). Then, the DMF solution of HBH (25 wt%) was added dropwise into the prepolymer at 80 °C under dried argon atmosphere (molar ratio of –NCO/–OH was controlled at 1.05). After that, the reaction was allowed to proceed at the same temperature for about 3.5 h until the NCO peak (~2270 cm^−1^) in the FT-IR spectrum disappeared completely. Subsequently, the solution was diluted to about 5 wt% and precipitated in cold diethyl ether. The product was dried to a constant mass at 40 °C under reduced pressure to obtain the white filiform PEEU.

The chemical structure of PEEU was characterized by ^1^H NMR (Figure S2), FT-IR (Figure S3) and GPC. ^1^H NMR (400 MHz, CDCl_3_, ppm): δ 5.10–5.31 (CH_3_–CH–), 4.80 (–NHCO–), 4.06–4.23 (–COOCH_2_–), 3.65–3.70 (–CH_2_–CH_2_O– of PEG), 3.15 (–NHCH_2_–), 2.29–2.41 (–CH_2_CO–), 1.67 (CH_3_–), 1.46–1.52 (–OCH_2_ (CH_2_)_3_CH_2_CO, –OCH_2_(CH_2_)_2_CH_2_O–), 1.34 (NHCH_2_(CH_2_)_4_CH_2_NH). FT-IR (ATR, cm^−1^): 3321 (N–H), 2937, 2864 (–CH_2_–), 1731 (C=O), 1687 (amide I), 1533 (amide II), 1091 (C–O–C of ester). GPC (THF): *M*_w_-GPC = 141,200, *M*_n_ = 101,300, *M*_w_/*M*_n_ = 1.39. 

### 2.3. Preparation of Poly(MPC-co-EHMA) (PMEH)

Monomers of EHMA (0.1 mol, 13.8 g) and MPC (0.025 mol, 7.4 g) were placed in a vacuum flask and the mixture was dissolved with ethanol (10 mL). After dried argon was bubbled into the solution to remove oxygen, AIBN (1 wt% of monomers) was added and the vacuum flask was sealed. The polymerization was carried out under vacuum at 60 °C for 12 h. The reaction mixture was cooled to room temperature, and then precipitated with a large amount of hexane. The precipitate was filtered off and dried under reduced pressure at room temperature. The copolymer was purified by Soxhlet extraction, first with water and then with ether, to remove the unreacted monomers. The reaction scheme is displayed in Figure S4.

The chemical structure of PMEH was characterized by ^1^H NMR (Figure S5), FT-IR (Figure S6), GPC and elemental analysis. ^1^H NMR (400 MHz, CDCl_3_, ppm): δ 4.52 (–COCH_2_CH_2_P–, –N^+^CH_2_CH_2_OP, –COCH_2_CH–), 3.65–3.83 (–N^+^CH_2_–, –N^+^CH_3_), 3.28 (–CH_2_–C–), 1.83 (–CH_2_CHCH_2_–), 1.28 (–CHCH_2_CH_3_, –CH(CH_2_)_3_CH_3_), 0.84 (CH_3_CH_2_–, CH_3_C–). FT-IR (ATR, cm^−1^): 3380 (–OH of H_2_O), 2957, 2928, 2863 (–CH_2_–), 1724 (C=O), 1238 (P=O), 1090 (C–O–C of ester), 1067 (P–O), 953 (–N^+^(CH_3_)_3_). GPC (THF): *M*_w_ = 27,000, *M*_n_ = 23,100, *M*_w_/*M*_n_ = 1.17. Anal. Calcd (%): C, 65.09; H, 10.21; O, 20.58; N, 1.29. Found: C, 65.12; H, 10.25; O, 20.52; N, 1.21.

### 2.4. Preparation of PEEU/PMEH Composite Films

The composite films were prepared by a solvent evaporation technique solvent evaporation technique according to Figure S7. Briefly, four weight percent solutions of both PEEU and PMEH solutions were prepared separately using ethanol/chloroform mixture (1/1 by volume) as solvent. The homogeneous solution containing the predetermined amounts of PEEU and PMEH (4.0 g/100 mL) was poured on to a Teflon mold, and the solvent was evaporated at room temperature for 72 h. Subsequently, the formed film was dried under reduced pressure for 24 h to eliminate the last traces of solvent. The PEEU/PMEH (PMPU) composite films were obtained with 0.25 ± 0.02 mm thickness. The chemical composition of PMPU films is listed in Table 1, and the PMPUs are described as PMPU-X (X: the weight percent of PMEH in the composites).

The chemical structure of PMPUs was characterized by FT-IR and the representative spectrum is shown in Figure S8. FT-IR (ATR, cm^−1^): 3324 (N–H), 2932, 2869 ((–CH_2_–), 1730 (C=O), 1683 (amide I), 1535 (amide II), 1240 (P=O), 1086 (C–O–C of ester), 1059 (P–O), 957 (–N^+^(CH_3_)_3_).

### 2.5. Characterization and Instruments

Characterization: ^1^H NMR spectra were recorded on a 400 MHz Avance II spectrometer (Bruker, Rheinstetten, Germany) using CDCl_3_ as solvent. FT-IR spectra were recorded between 4000 and 400 cm^−1^ on a Bruker Alpha infrared spectrometer (Bruker, Rheinstetten, Germany) equipped with a Bruker platinum ATR accessory. The weight average molecular weight (*M*_w_), number average molecular weight (*M*_n_) and polydispersity index (*M*_w_/*M*_n_) of polymers were measured by gel permeation chromatography (GPC) on a Water Alillance GPC 2000 system (Waters, Milford, MA, USA) with tetrahydrofuran as the eluting solvent. Elemental analysis (C, H, N, and O) was performed on Elementar Vario E1 III analyzer (Elementar, Langenselbold, German).

Thermogravimetric analysis (TGA): TGA tests were conducted using a TGA 2050 analyzer (Universal, New Brunswick, NJ, USA). The mass loss of the dried samples was performed under nitrogen inert atmosphere (N_2_, 40 mL/ min) from 50 to 600 °C at a heating rate of 15 °C/min.

Tensile properties: According to the national standard GB/T1040-2006, tensile stress–strain tests were measured using a single-column tensile test machine (Model HY939C, producer, Dongguan, China) at room temperature with a cross-head speed of 50 mm/min. The films were cut in a dumbbell shape with neck width of 4.0 mm and length of 30 mm, respectively. For each data, the result was the average value of five parallel measurements. 

Water absorption: The amount of absorbed water by the film is the parameter to evaluate the water-swelling property of the films. The preweighed dry film disks (*m*_o_) with ~10 mm diameter were immersed in deionized water at 37 ± 0.1 °C and equilibrated for about 48 h. The swollen samples were blotted with laboratory tissue to remove the surplus absorbed water, and weighed immediately (*m*_t_). The water-swelling property of the film was expressed as the water absorption calculated from the following formula. Water absorption (%) = (*m*_t_ − *m*_o_)/*m*_o_ × 100. The results reported were the average values for at least five replicated samples.

Water contact angle: The surface hydrophilicity was assessed by measuring the water contact angles formed between the water drops and the film surface. The sessile static water contact angles against the film surface were measured by a contact angle setup of KSV CAM 200 (KSV Instruments, Helsinki, Finland) using a sessile drop method at room temperature. Before measurement, the film specimens were immerged in distilled water for 2 h and then dried under vacuum. To ensure that the droplets did not penetrate the compact material, the test was carried out within 5 s. The test was performed on six samples, and three drops were applied on each sample.

In vitro degradation: Film degradation was quantified by the weight loss in PBS (pH = 7.4). The film in a disk shape (diameter: 10 mm) was placed into a sealed bottle which containing 10 mL PBS solution, and incubated at the temperature of 37 ± 0.1 °C. At given time intervals, the sample was removed from the solution, washed with distilled water and dried in a vacuum oven at 25 °C until constant weight. The weight loss was calculated using the following equation to evaluate the films degradation. Weight loss (%) = (*W*_o_ − *W*_r_)/*W*_o_ × 100, where *W*_r_ is the rest weight of the sample after degradation for a predetermined time and *W*_o_ is the weight of the dry sample. The tests were carried out until the films lost tensile properties and became fragments. Each test was repeated at least three times and the results were the average.

Surface morphologies: The samples after in vitro degradation for a fixed time period were collected to observe the surface morphologies. The dried samples were coated with gold for the morphological observation by using a SU8010 FE-SEM (Hitachi, Tokyo, Japan).

Protein adsorption: The Bradford protein determining method was used to determine the amount of adsorbed protein onto the film surface when using bovine serum albumin (BSA, Shanghai Aladdin Reagent Co., Ltd., Shanghai, China) [38,39] and human plasma fibrinogen (HPF, Shanghai Macklin Biochemical Co., Ltd., Shanghai, China) [40,41] as model proteins. The film discs (~10 mm diameter) were equilibrated with PBS (pH = 7.4) for ~12 h to achieve complete hydration, and then immersed in a solution of 1.0 mL protein solution (BSA: 45 µg/mL; HPF: 30 µg/mL) for 3 h at the temperature of 37 ± 0.5 °C. The discs were gently taken off and rinsed sufficiency with PBS to remove the unbound BSA. After sonication in sodium dodecylsulfonate aqueous solution (1 wt%) for 30 min to detach the adsorbed protein on the surface, a micro-Bradford protein analysis kit (Sangon Biotech Co., Ltd., Shanghai, China) with a multiwall microplate reader (Multiskan Mk3-Thermolabsystems, Thermo Fisher Scientific, Inc., USA) was used to determine the concentration of the adsorbed BSA and HPF in the solutions at 595 nm and 562 nm, respectively. The amount of proteins adsorbed on the surface could be calculated from the protein concentration in the solution. At least three replicate samples were tested to ensure reproducibility of the measurements, and values relative to the controls (PBS) were collected.

Platelet adhesion: To evaluate the interactions between blood and films, the platelet adhesion tests were performed in this study. Platelet-rich plasma (PRP) was obtained from fresh rabbit blood (Shandong Success Biotechnology Co., Ltd., Jinan, China) by centrifugation of blood in a sodium citrate buffer at 2000 rpm for 20 min at 4 °C. The disk-shaped samples (diameter: 10 mm) were contacted with PBS (pH = 7.4) for 2 h to equilibrate the surface, and then removed from the solution and incubated with 1.0 mL PRP at 37 °C for 1 h. The samples were rinsed thoroughly with fresh PBS to remove nonadherent platelets. The platelets adhering to the surface were fixed with 2.5% glutaraldehyde for 30 min at 40 °C. Then, the discs were dehydrated by treating with gradual ethanol/water solutions (60, 70, 80, 90, 100% (*v*/*v*)) for 30 min in each step and allowed to dry at room temperature on a clean bench. Finally, the platelet-attached surfaces were coated with gold prior to observation by FE-SEM on different fields of surfaces.

## 3. Results and Discussion

### 3.1. Thermal Stability

**Figure 1:**
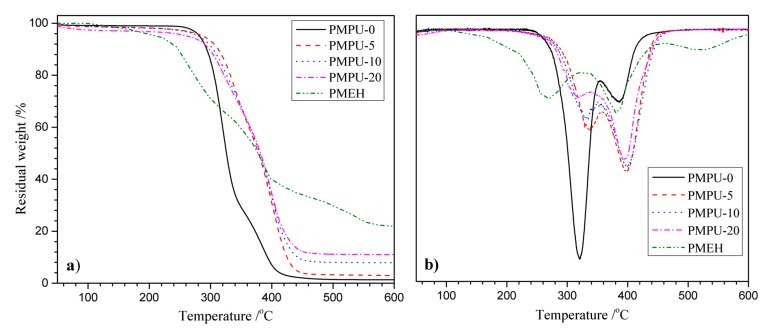
(**a**) Thermogravimetric analysis (TGA) and (**b**) differential gravimetric thermal analysis (DTGA) curves of composite films with varying PMEH content.

TGA analysis is often used to evaluate the thermal stability of materials; Figure 1 shows the TGA and differential thermal gravimetric analysis (DTGA) curves of the composite films with varying PMEH content. There were two clear consecutive weight losses observed in curve of pure PEEU (PMPU-0). The first weight loss occurred at ~247–353 °C with the maximum decomposition temperature (*T*_max_) of 320 °C and the weight loss was 68 wt%, which was attributed to the decomposition of carbamate and ester bonds. Another weight loss occurred at a higher temperature of 354–438 °C; *T*_max_ of 385 °C was assigned to the decomposition of ether bonds. The remaining weight was lower than 2 wt%, indicating that the PMPU-0 decomposed almost completely. While the PMEH exhibited obvious three-step weight loss, which was due to the complex structure. The initial decomposition temperature (~200 °C) was lower that of pure PEEU, but the PMEH had higher thermal stability than pure PEEU at high-temperature region (above 400 °C). The residue weight was more than 20 wt%, which should be ascribed to the nitrogen and phosphonium salt formed after the decomposition of PMEH segments. Compared with PEEU, the thermal stability of composite films (PMPU-5~PMPU-20) increased obviously and the *T*_max_ was ~20 °C higher than that of pure PEEU, which could be due to the interaction (maybe H bonds) among the chains of PMEH and PEEU. Obviously, the increasing residue weight should be attributed to the increase of PC content in composite films. No other weight loss steps found in the curves of PMPU films indicated that the PMEH component was compatible with PEEU component.

### 3.2. Tensile Properties

**Figure 2:**
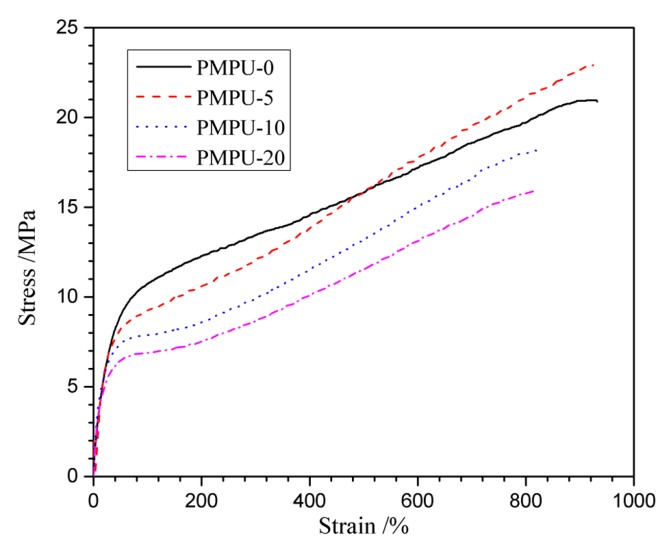
Stress–strain behaviors of PMPU films with varying PMEH content.

Tensile property was an important quality for long-term implant biomaterials. The typical stress–strain curves of the composite films with varying PMEH content are shown in Figure 2, and the corresponding characteristic values obtained from the curves are listed in Table 2. From the curves, it could be found that the composite films with PMEH content increasing from 0 to 20 wt% (PMPU-0~PMPU-20) behaved as soft elastic materials, displaying a smooth transition from the elastic to plastic deformation regions [42]. The pure PEEU (PMPU-0) containing uniform-size hard segments exhibited good tensile properties with ultimate stress of 20.8 MPa, strain at break of 930% and initial modulus of 19.5 MPa, which was due to the compact physical-linking network structure formed by denser H bonds existing not only among carbamate groups but between carbamate and ether/ester groups [43]. When the PMEH was blended in PEEU, the strain at break decreased gradually. However, with the PMEH content increasing from 5 to 20 wt%, the ultimate stress of the composites films first expressed no obvious change and then decreased gradually. When the PMEH content in composites is as low as 5 wt% (PMPU-5), the PMEH can be homo-dispersed in PEEU and acts as a filler; additionally, the PMEH content maybe too low to be reflected, the two reasons result in almost unchanged strain at break and ultimate stress. When the PMEH content is higher than 5 wt% (PMPU-10 and PMPU-20), the residual brittle PMEH can be aggregated as a stress concentration point and destroy the tensile properties, thus the strain at break, ultimate stress and initial modulus of the composite films decrease [44]. However, the composite film with high PMEH content up to 20 wt% (PMPU-20) exhibited good tensile properties with strain at break of 820% and ultimate stress of 15.8 MPa, which could also meet the clinical requirements of long-term implant medical biomaterials, such as cartilage.

### 3.3. Surface and Bulk Hydrophilicity

**Figure 3:**
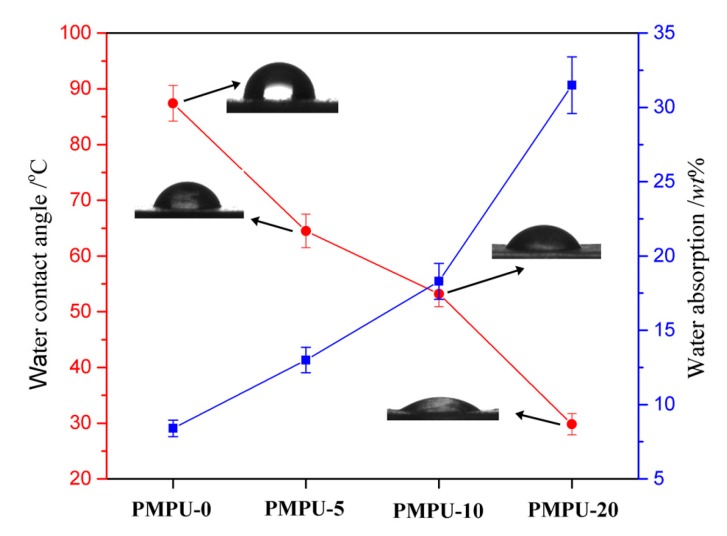
Water contact angle and water absorption of composite films with varying PMEH content.

The surface and bulk hydrophilicity of the composite films with varying PMEH content were characterized by measuring the surface water contact angle and water absorption, and the results are displayed in Figure 3. The pure PEEU film exhibited a hydrophobic surface with the water contact angle of 87.4°, and the water contact angle showed a trend to decline dramatically after PMEH was introduced. With the PMEH content varying from 5 to 20 wt% (PMPU-5~PMPU-20) in composite films, the water contact angle decreased dramatically from 64.5° to 29.8°, presenting an increasingly hydrophilic surface. After water immersion of the composite films, the hydrophilic PC units can rearrange to the water-side and the concentration of the PC units on the surface increases. The zwitterionic PC groups interact with water molecules by hydrogen bonding, which forms a hydration layer on the film surface and improves the surface hydrophilicity [45]. The equilibrium water absorption of the composite films, which was reached after immersion in water for 48 h, increased gradually from 8.4 to 31.5 wt% with the PMEH content varying from 0 to 20 wt% (Figure 3). It is also ascribed to the hydrophilicity of PC units which can bind much water into film. The results manifested that the surface and bulk hydrophilicity, an important role in the hydrolytic degradation and surface hemocompatibility, could be affected by the content of hydrophilic PC units in composites.

### 3.4. In Vitro Degradation

**Figure 4:**
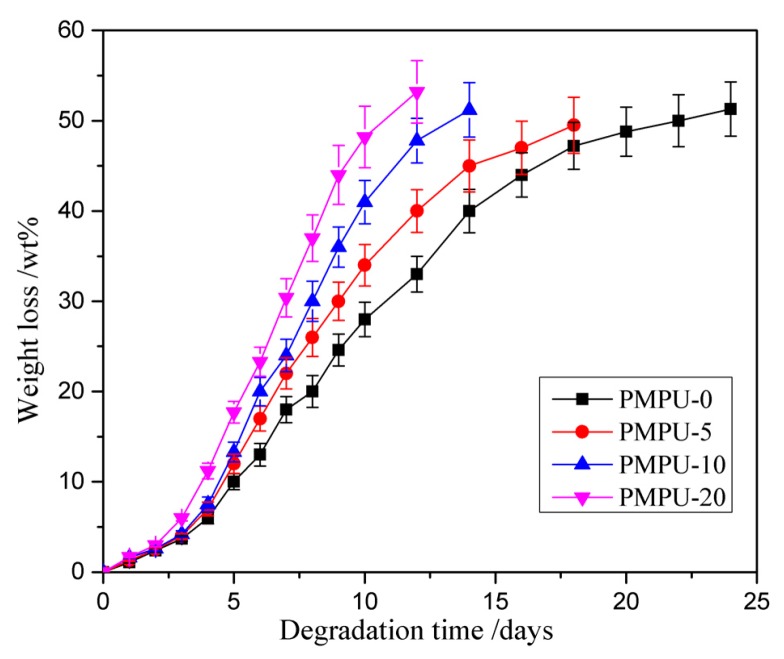
Weight loss curves of composite films with varying PMEH content in PBS (pH = 7.4) at 37 ± 0.1 °C.

In vitro hydrolytic degradation was examined by the weight loss in PBS solution at 37 °C, and the degradation behaviors of composite films are shown in Figure 4. The degradation process included three stages. In the first several days, all films exhibited a similar degree of degradation with slight weight loss less than 10 wt%, which should be attributed to the hydration and swelling of the films in the incipient stage. After that, the weight loss rate increased sharply, mainly because the films were cleaved to water-soluble molecules that were soluble in the media. Finally, the films become fragments and lost the tensile properties. In addition, the degradation rate of composite films increased with the increase of PMEH content, and the time taken for PMPU-0, -5, -10, and -20 films to become fragments was ~24, 18, 14, and 12 days, respectively. As the description in test of water absorption, high hydrophilic PC units can bind more water molecules, which make the ester groups in PEEU chains easily expose to water molecules. Thus, the chain scission occurs easily through hydrolysis of ester bonds, which leads to an increased degradation rate. From the results, it can be found that the degradation rate of composite films can be adjusted by changing the PMEH content. 

**Figure 5:**
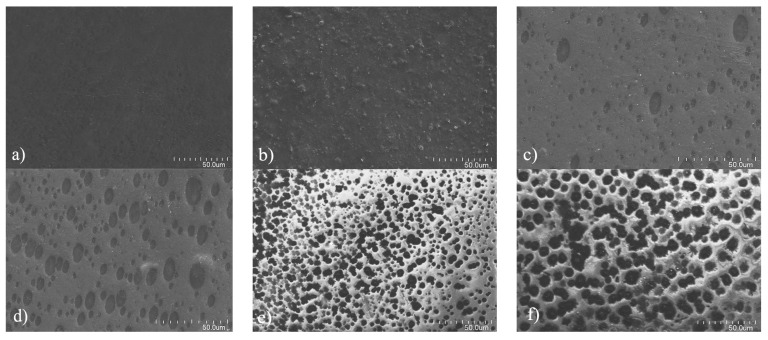
Surface morphologies of PMPU-10 film in PBS (pH = 7.4) at 37 ± 0.1 °C after (**a**) 0, (**b**) 3, (**c**) 5, (**d**) 7, (**e**) 10, and (**f**) 12 days of degradation.

The degradation process can be directly reflected by morphological changes in film surface. The typical surface morphologies of PMPU-10 after different degradation periods were presented in Figure 5. The nondegraded film (Figure 5a) was semitransparent with a relatively smooth surface. A rough surface was observed when the film was degraded for 3 days (Figure 5b), and more and more irregular cavities appeared on the film surface with the further degradation (Figure 5c,d). A large number of holes were observed after 10 and 12 days of degradation (Figure 5e,f), which indicates the film gradually losing its tensile properties. 

### 3.5. Protein Adsorption

**Figure 6:**
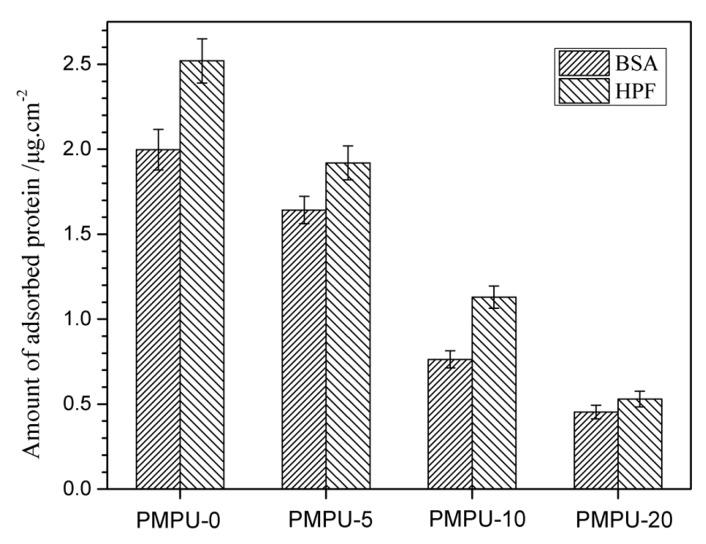
The amount of adsorbed protein on the surface of composite films with varying PMEH content at 37 ± 0.5 °C.

The protein adsorption on biomaterials’ surfaces has been considered the first step to test many undesired biological reactions [46], and the formation of thrombus at the interface is dependent on the number and state of the protein adsorption layer [47]. The adsorption behaviors of protein (BSA and HPF) on the composite film surface are exhibited in Figure 6. As shown in Figure 6, the amount of adsorbed protein on pure PEEU (PMPU-0) film surface was 1.99 μg/cm^2^ for BSA and 2.52 μg/cm^2^ for HPF, both decreased significantly after the introduction of PMEH into the films. With the PMEH content increasing from 5 to 20 wt%, the absorbed amount of BSA and HPF on the composite film surface decreased gradually from 1.65 to 0.45 μg/cm^2^ and from 1.92 to 0.53 μg/cm^2^, respectively. It may be ascribed to the presence of a hydrated layer around the zwitterionic PC groups on the film surface which reduces molecular interactions with protein [48]. In addition, the proteins are very difficult to replace the water molecule because of the strong interaction between water molecules and PC groups [27]. The lower protein adsorption capacity of composite films means better surface hemocompatibility.

### 3.6. Platelet Adhesion

**Figure 7:**
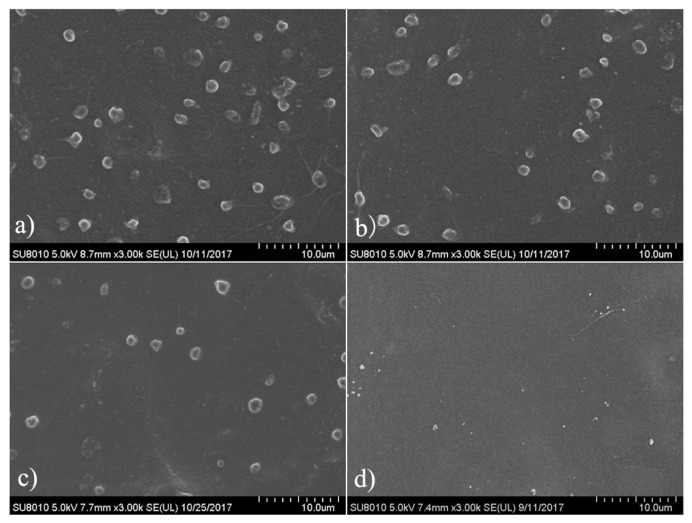
Representative SEM images of platelet adhesion on the surface of (**a**) PMPU-0, (**b**) PMPU-5, (**c**) PMPU-10, and (**d**) PMPU-20 films.

The platelet adhesion on materials surface is an effective and frequently used measurement to evaluate the hemocompatibility of blood-contacting biomaterials [49]. The platelet adhesion on the composite films after contacting with rabbit PRP was assessed by SEM observation, and the representative micrographs are given in Figure 7. Massive platelets adhered on the surface of pure PEEU film (PMPU-0, Figure 7a). Some of the platelets aggregated to some extent and some exhibited deformation and formed pseudopods, presenting the highly activated state. The platelet adhesion on the surface was effectively suppressed after PMEH was introduced to the PEEU, and moreover, deformation of adherent platelets was also hindered on the composite film surface. When the composition of PMEH in the composites was 20 wt% (PMPU-20, Figure 7d), very few adherent platelets were observed, which proved an excellent antiplatelet adhesion surface. It has been reported that PC groups on the surface can migrate and reorientate onto the interface between the film surface and water, which forms a mimetic structure of cell outer membrane and possesses outstanding antiplatelet adhesion property [50,51]. Although the results of platelet adhesion test demonstrated that the films surface hemocompatibility was improved dramatically by adding a small amount of the additional of PMEH, the surface hemocompatibility need further evaluations, such as using human whole blood and subcutaneous implant tests.

## 4. Conclusions

In this paper, the novel composites of the biodegradable PEEU and blood-compatible PC copolymer (PMEH) were prepared by blending from a homogeneous solution, and the corresponding films were obtained by a solvent evaporation method. The physicochemical properties of the composite films with varying PMEH content were studied. The composite films (PMPU) exhibited higher thermal stability than that of original PEEU film. With PMEH content increasing from 5 to 20 wt%, the PMPU films also possessed satisfied tensile properties with ultimate stress of 22.9–15.8 MPa and strain at break of 925–820%. The surface and bulk hydrophilicity of PMPU films were closely related to the content of hydrophilic PC groups. In vitro degradation studies indicated that the degradation rate increased with the increment of PMEH content, and the time of the composite films becoming fragments was 12–24 days. The reduction of protein adsorption and platelet adhesion on the composite films surface manifested good surface hemocompatibility. Due to the biodegradability, satisfied tensile properties and good surface hemocompatibility, the composites can be expected to be applied in blood-contacting implant materials. 

## Figures and Tables

**Table 1:** The chemical composition of PEEU/PMEH (PMPU) films.

	Films	PMPU-0	PMPU-5	PMPU-10	PMPU-20
Components					
PEEU/g		4.0	3.8	3.6	3.2
PMEH/g		0	0.2	0.4	0.8
PMEH content/wt%		0	5	10	20

**Table 2:** Tensile properties of PMPU films with varying PMEH content.

Films	Strain at Break (%)	Ultimate Stress (MPa)	Yield Stress (MPa)	Yield strain (%)	Initial Modulus (MPa)
PMPU-0	932 ± 41	20.8 ± 2.4	9.97 ± 1.2	51.1 ± 4.2	19.5
PMPU-5	925 ± 38	22.9 ± 2.3	8.15 ± 1.05	43.3 ± 3.3	18.8
PMPU-10	825 ± 32	18.2 ± 1.9	7.26 ± 0.92	41.3 ± 3.4	17.5
PMPU-20	820 ± 34	15.8 ± 1.4	6.20 ± 0.76	39.8 ± 3.0	15.6

## Supplementary Materials

The supplementary materials are available online at https://www.mdpi.com/2073-4360/11/5/860/s1.
