# Supplementary material for: Preparation, Physicochemical Properties, and Hemocompatibility of the Composites Based on Biodegradable Poly(Ether-Ester-Urethane) and Phosphorylcholine-Containing Copolymer
Source: Polymers (Basel). 2019 May 11;11(5):860. doi: 10.3390/polym11050860 (PMC6572198; doi:10.3390/polym11050860)
Supplement: Supplementary file 1 [file polymers-11-00860-s001.pdf]

Supporting Information

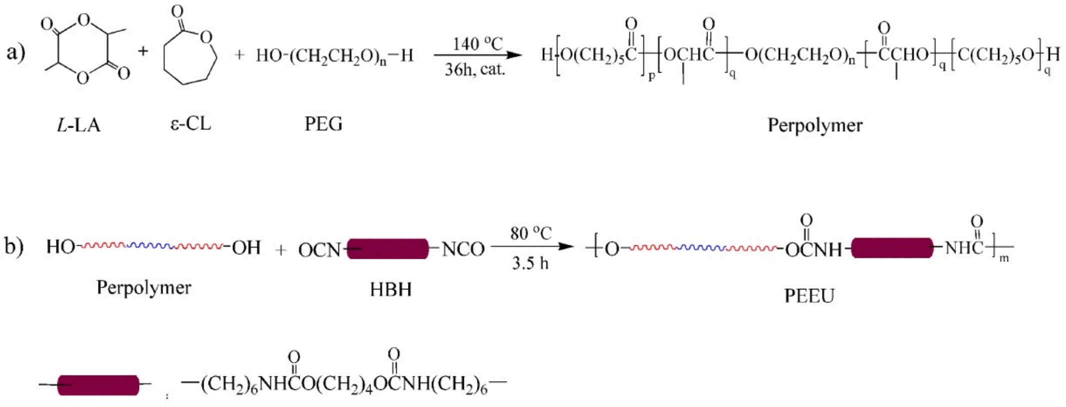

Figure S1. The reaction scheme of PEEU

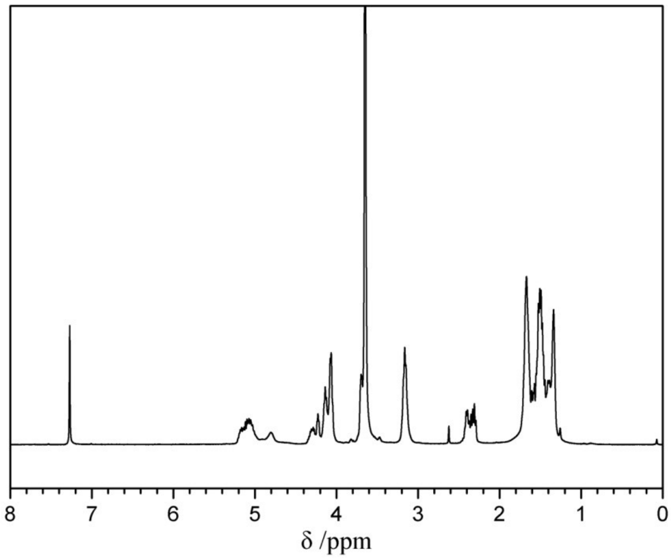

Figure S2. ^{1}H NMR spectrum of PEEU

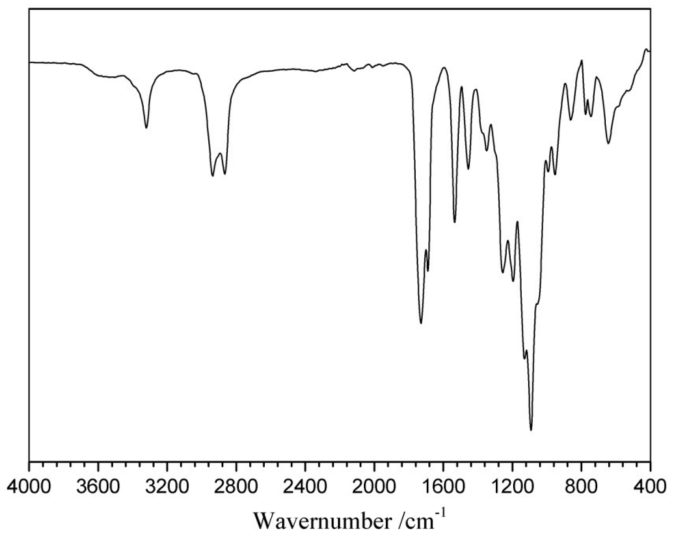

Figure S3. FT-IR spectrum of PEEU

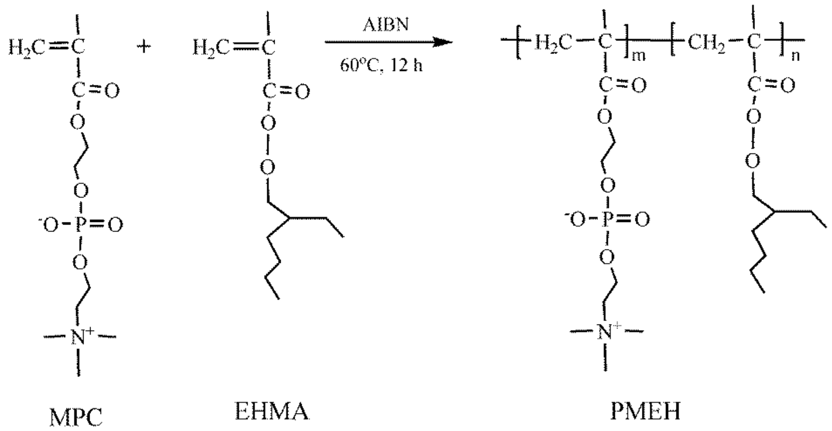

Figure S4. The reaction scheme of PMEH

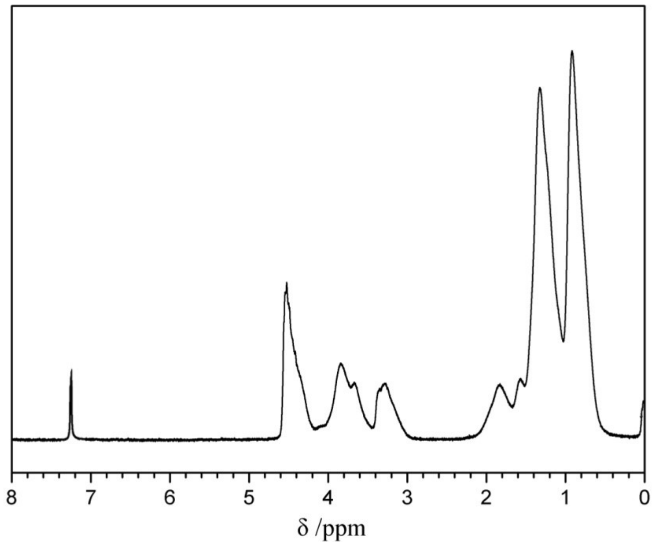

Figure S5. ^{1}H NMR spectrum of PMEH

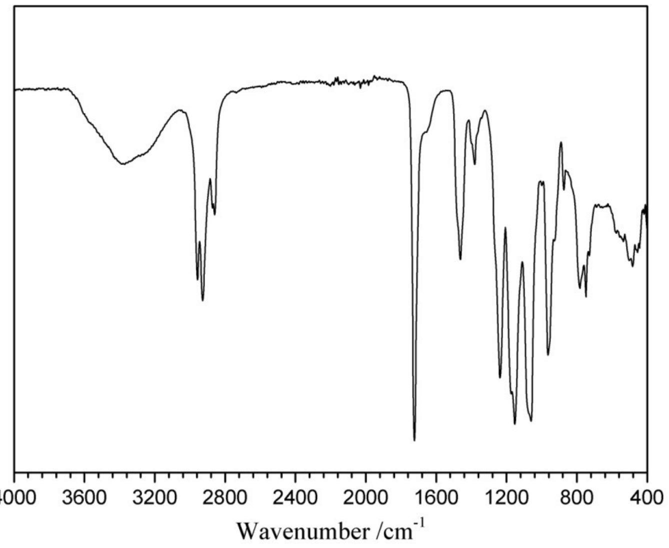

Figure S6. FT-IR spectrum of PMEH

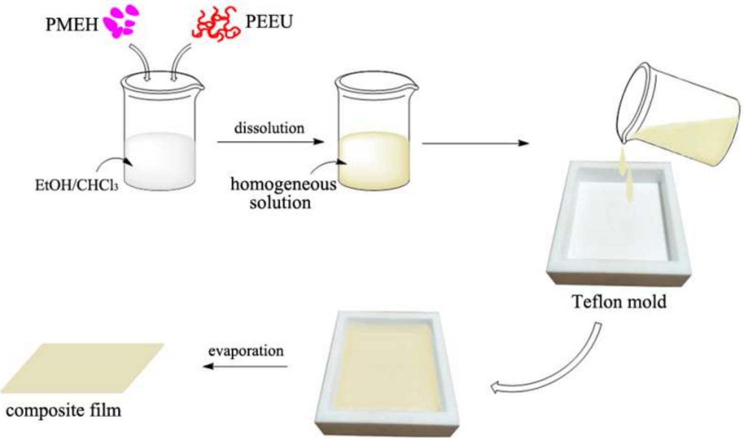

Figure S7. Preparation of composite film

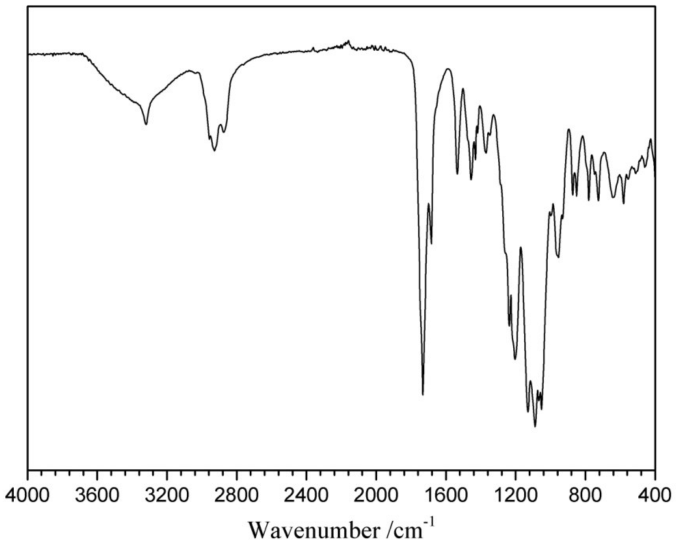

Figure S8. FT-IR spectrum of PMPU-10
